# Effects of lower limb and pelvic pin positions on leg length and offset measurement errors in experimental total hip arthroplasty

**DOI:** 10.1186/s13018-021-02347-z

**Published:** 2021-03-16

**Authors:** Haruo Kawamura, Yasuhiko Watanabe, Tomofumi Nishino, Hajime Mishima

**Affiliations:** 1 Department of Orthopaedic Surgery, Kenhoku Medical Center Takahagi Kyodo Hospital, 1006-9 Kamiteduna Agehochou, Takahagi, Ibaraki, 318-0004 Japan; 2 Department of Orthopaedic Surgery, Ryugasaki Saiseikai Hospital, 1-1 Nakasato, Ryugasaki, Ibaraki, 301-0854 Japan; 3 Department of Orthopaedic Surgery, Institute of Clinical Medicine and University Hospital, University of Tsukuba, 1-1-1 Tennodai, Tsukuba, Ibaraki, 305-8575 Japan

**Keywords:** Leg length, Offset, Total hip arthroplasty, Calliper, Limb position, Iliac pin position

## Abstract

**Background:**

Leg length (LL) and offset (OS) are important factors in total hip arthroplasty (THA). Because most LL and OS callipers used in THA depend on fixed points on the pelvis and the femur, limb position could affect measurement error. This study was conducted on a THA simulator to clarify the effects of lower limb position and iliac pin position on LL and OS errors and to determine the permissible range of limb position for accurate LL and OS measurement.

**Methods:**

An LL and OS measurement instrument was used. Two pin positions were tested: the iliac tubercle and the top of the iliac crest intersecting with the extension of the femoral axis. First, the limb was moved in one direction (flexion-extension, abduction-adduction, or internal-external rotation), and LL and OS were measured for each pin position. Next, the limb was moved in combinations of the three directions. Then, the permissible range of combined limb position, which resulted in LL and OS measurement error within ±2 mm, was determined for each pin position.

**Results:**

Only 4° of abduction/adduction caused 5–7 mm error in LL and 2–4 mm error in OS, irrespective of pin position. The effects of flexion–extension and internal–external rotation on LL error were smaller for the top of the iliac crest than for the iliac tubercle, though OS error was similar for both pin positions. For LL, the permissible range of the combined limb position was wider for the top of the iliac crest than for the iliac tubercle.

**Conclusion:**

To minimize LL and OS measurement errors in THA, adduction–abduction must be maintained. The iliac pin position in the top of the iliac crest is preferred because it provides less LL measurement error and a wider permissible range of combined limb position for accurate LL measurement.

## Introduction

Total hip arthroplasty (THA) has revolutionized patient care in end-stage hip arthritis cases. In order to achieve an optimal functional result after THA, it is desirable to properly adjust leg length (LL) and restore offset (OS) within the hip. LL inequality after THA can cause limping, pain in the knee or back, abnormal force transmission across the hip, revision surgery, and even litigation [1–7]. Inadequate OS restoration can also cause hip-joint instability, increased polyethylene wear, and decreased range of motion [8, 9].

During THA, Charnley first advocated the use of a femoral component with an extended offset, though it was not generally adopted. The high-offset neck option was introduced to the orthopaedic field in the late 1990s, and it soon became generally used in the early 2000s [8, 10]. Surgeons wishing to increase femoral neck OS during THA now had three options:(1) increasing the neck length of the modular femoral head, (2) using a stem with a lower neck–shaft angle, or (3) using a femoral stem with a constant neck–shaft angle, but a medialized ‘high-offset’ femoral neck. Options (1) and (2) increase OS, but also alter LL, whereas option (3) only affects OS and not LL. Since surgeons wanted to manage both LL and OS more precisely, several LL/OS callipers were developed [6, 11, 12].

The LL and OS measurement instrument (LOMI; Smith & Nephew, Memphis, TN, USA) is a unique device to simultaneously measure both LL and OS during THA [10]. The LOMI detects changes in LL and OS (i.e. global offset) prior to hip dislocation and after the trial components or final implants have been inserted. The use of the LOMI increases operation time by 5 min compared to unassisted surgery. In many cases of primary osteoarthritis without marked deformity, LL and OS remain unchanged after THA. On the other hand, in secondary osteoarthritis, such as developmental dysplasia of the hip (DDH), the anatomical implantation of the prostheses might change the LL and OS greatly from the pre-operative condition. Therefore, to adjust LL properly and restore OS according to pre-operative planning, it would be helpful to be able to measure the change in LL and OS during THA. Because the neck–shaft angles of the current femoral stems are around 130°, a 3-mm neck-length change by modular heads results in approximately 2-mm changes in LL and OS. Therefore, accuracy of intra-operative LL and OS measurement should be less than 2 mm. Furthermore, limb position repeatability that results in no more than a 2-mm leg length and/or offset measurement error is considered to be a prerequisite for leg length and offset measurement. As with other LL callipers, intra-operative measurements with the LOMI are based on measurements between fixed points on the pelvis and femur. Because the fixed reference points are located away from the centre of the hip joint, small errors in femur repositioning can lead to large errors in measurement [6]. It is not easy to place the femur in exactly the same position between measurements during THA. Additionally, LL measurements are known to be affected by the location of the fixed point on the pelvis [12], and the errors of LL and OS measurements due to the lower limb position in relation to iliac pin position have not been clearly described. Therefore, this study was conducted on a THA simulator to clarify the effects of lower limb position (flexion (FX)/extension (EX), abduction (ABD)/adduction (ADD), and internal rotation (IR)/external rotation (ER)) on LL and OS errors in relation to iliac pin position. Furthermore, the permissible range of combined limb position, which made LL and OS measurement errors within ±2 mm, was determined.

## Materials and methods

**Fig. 1:**
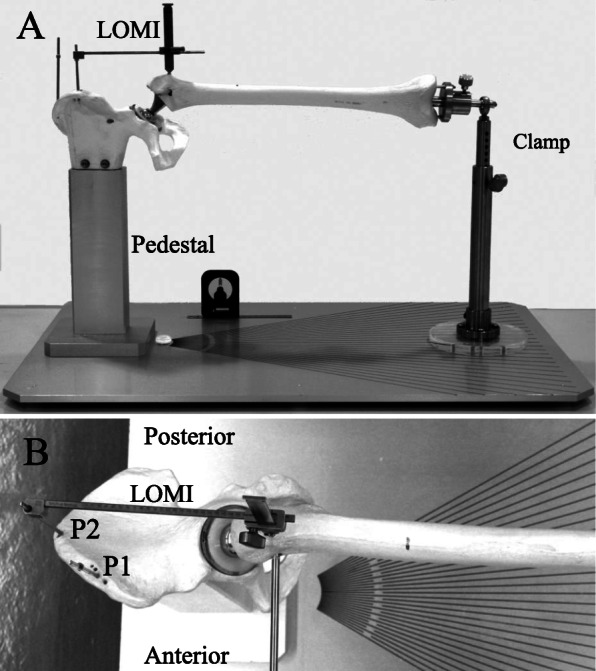
Photographs of the experimental device simulating total hip arthroplasty in the lateral decubitus position. **a** Anterior–posterior view. A leg length and offset measurement instrument (LOMI) is mounted on a pelvic pin inserted in the iliac tubercle (P1). **b** Lateral view. The LOMI is mounted on top of the iliac crest intersecting with the extension of the femoral axis (P2). P1 is situated 22° anterior in relation to the centre of rotation of the hip

A custom-made experimental device was developed to simulate the thigh and pelvis in the lateral decubitus position as they would present during THA surgery (Fig. 1). A Sawbone hemi-pelvis model was rigidly fixed to a heavy pedestal on a solid metal baseboard (width 50 cm, length 90 cm, and thickness 2 cm). A 56-mm acetabular metal shell and an appropriate polyethylene liner (Reflection SP3 and XLPE, Smith & Nephew) were firmly fixed to the hemi-pelvis in the usual surgical fashion. A #12 femoral broach of the Synergy hip system (Smith & Nephew) was tightly seated into the Sawbone femur using the usual femoral preparation. A high-offset trial neck with a 28-mm diameter, cobalt-chromium femoral head (+0 neck length) was attached to the femoral broach. A bar was fixed to the hole at the shoulder of the broach as an indicator of rotation. The bar was perpendicular to the long axis of the neck-shaft of the broach. The distal end of the femur was held with a custom-made clamp, which could change the angles of the hip joint three-dimensionally as desired. The centre of rotation of the hip joint was projected on the baseboard to represent the origin of a protractor for FX/EX. Next, to define the 0° FX/EX angle, a line parallel to the longer edge of the base board was drawn from the origin. Then, lines were drawn on the base board from 30° FX to 30° EX in 2° increments. The IR/ER angle was determined using a level gauge on the bar. When the bar was horizontal, IR/ER was defined as being 0°. Similarly, the ADD/ABD angle was determined using the level gauge on the lateral surface of the femur. When the femur was horizontal, ADD/ABD was defined as being 0°. Two reference pins were set in the ilium (Fig. 1b). The first pin was put in the iliac tubercle (P1). The iliac tubercle, a prominence of the iliac wing, is situated just posterior to the anterior–superior iliac spine. P1 is usually chosen in actual THA to achieve secure pin fixation [8, 10]. P1 is situated 22° anterior in relation to the centre of rotation of the hip. The second pin was put in the top of the iliac crest that would intersect with the long axis of the femur at the 0° FX/EX line (P2). As for the measuring point, the lateral prominence of the greater trochanter was chosen. To clearly mark the point, a 3.5-mm cortical screw was placed.

We conducted a preliminary experiment to examine the accuracy and repeatability of the LOMI in the experimental THA system. The pelvic pins were set in P1 and P2. The femur was seated in neutral (0° of FX/EX, ABD/ADD, and IR/ER) position. Using three different modular heads (neck length; − 5, 0, and 5 mm), LL and OS were measured three times on two different days. Mean values and standard deviations were calculated for all measurements. Because the neck-shaft angle of the Synergy stem is 131°, a 5-mm difference in the neck results in a 3.3-mm difference in leg length and a 3.8-mm difference in offset. For P1, the mean value and standard deviation were 3.5 ± 0.7 mm in LL and 3.5 ± 0.4 mm in OS, respectively. For P2, the mean value and standard deviation were 3.3 ± 0.5 mm in LL and 3.5 ± 0.9 mm in OS, respectively. Therefore, the accuracy and repeatability of the LOMI on the THA simulator were found to be within 0.3 and 0.9 mm, respectively.

To determine LL and OS measurement errors associated with limb position, the limb position was changed. Initially, it was changed in one direction while the other directions were fixed. The range of limb position change was 30° FX to 30° EX, 18° ABD to 10° ADD, and 30° IR to 30° ER, each in 2° increments.

Next, to determine the permissible range of the combined limb position for accurate LL and OS measurement, the position was changed with various combinations of ABD/ADD, FX/EX, and IR/ER to simulate the actual surgical situation. LL and OS measurement errors were set within ±2 mm for the permissible ranges. Tested angles in ABD/ADD were 0° and 4° of ADD. At each ADD angle, FX/EX was changed from 20° FX to 8° EX in 4° increments. Furthermore, IR/ER was changed from 20° IR to 20° ER in 4° increments at each FX/EX angle. Thus, a total of 88 measurements were done at each ADD angle. A chi-square test was used to compare the permissible range for P1 and P2. *P* < 0.05 was considered statistically significant.

The pelvic pins were set in P1 and P2. For each limb position, LL and OS were measured three times, and the mean values were calculated. The differences of the mean values of LL and OS between neutral (0° of FX/EX, ABD/ADD, and IR/ER) and each position were defined as the errors. Error of the limb position in one direction is shown by line graphs, and error in the combined directions is shown by contour graphs. Microsoft Excel for Microsoft 365 MSO was used for graphs.

## Results

### Leg length and offset errors associated with unidirectional change of limb position

**Fig. 2:**
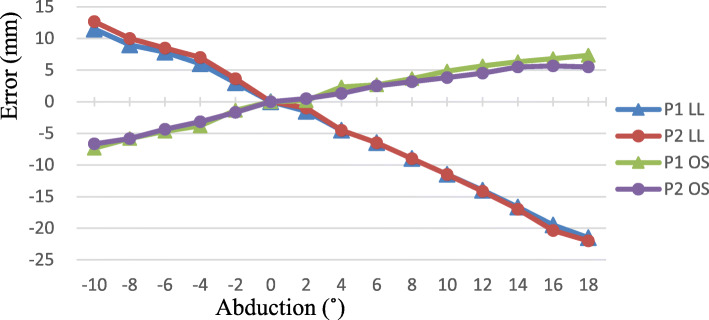
Leg length and offset measurement errors in relation to abduction–adduction. The limb is moved from 18° abduction to 10° adduction. P1, pelvic pin position in the iliac tubercle; P2, pelvic pin position in the iliac crest in line with the longitudinal axis of the femur; LL, leg length; OS, offset

The effects of ADD/ABD on LL and OS errors were enormous, irrespective of pin position (Fig. 2). As ADD increased, LL became longer and OS became smaller. Conversely, as ABD increased, LL became shorter and OS became larger. ADD/ABD of only 4° caused approximately 5–7-mm error in LL and 2–4-mm error in OS. The total error was as much as 34 mm in LL and 14 mm in OS.

**Fig. 3:**
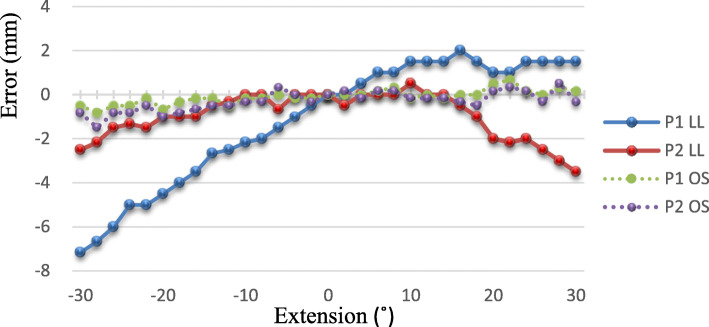
Leg length and offset measurement errors in relation to flexion-extension. The limb is moved from 30° flexion to 30° extension. P1, pelvic pin position in the iliac tubercle; P2, pelvic pin position in the iliac crest in line with the longitudinal axis of the femur; LL, leg length; OS, offset

The effect of FX/EX on LL was larger for P1 than for P2 (Fig. 3). In P1, LL became shorter in FX and longer in EX; the total error was 9.2 mm. In P2, LL became slightly shorter either in FX or EX; the total error was 4 mm. The effect of FX/EX on OS was very small, within 1.5 mm for both P1 and P2.

**Fig. 4:**
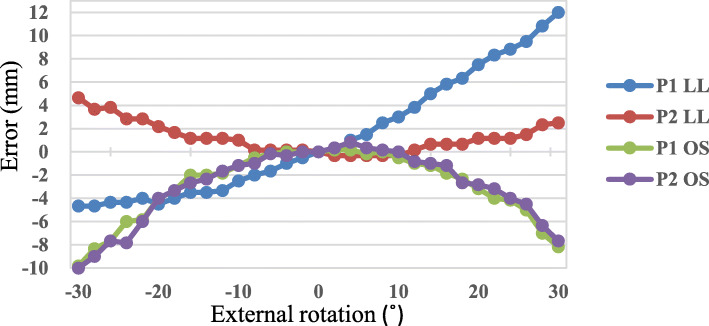
Leg length and offset measurement errors in relation to internal rotation–external rotation. The limb is moved from 30° internal rotation to 30° external rotation. P1, pelvic pin position in the iliac tubercle; P2, pelvic pin position in the iliac crest in line with the longitudinal axis of the femur; LL, leg length; OS, offset

The effect of IR/ER on LL was also larger for P1 than for P2 (Fig. 4). In P1, LL became shorter with an increase of IR and longer with an increase of ER; the total error was 17 mm. In P2, LL became slightly longer with an increase in either IR or ER; the total error was 5 mm. The effect of IR/ER on OS was similar for P1 and P2. OS became smaller with increases of IR and ER; the total error was 10 mm.

### The permissible range of combined limb position for accurate leg length and offset measurements

**Fig. 5:**
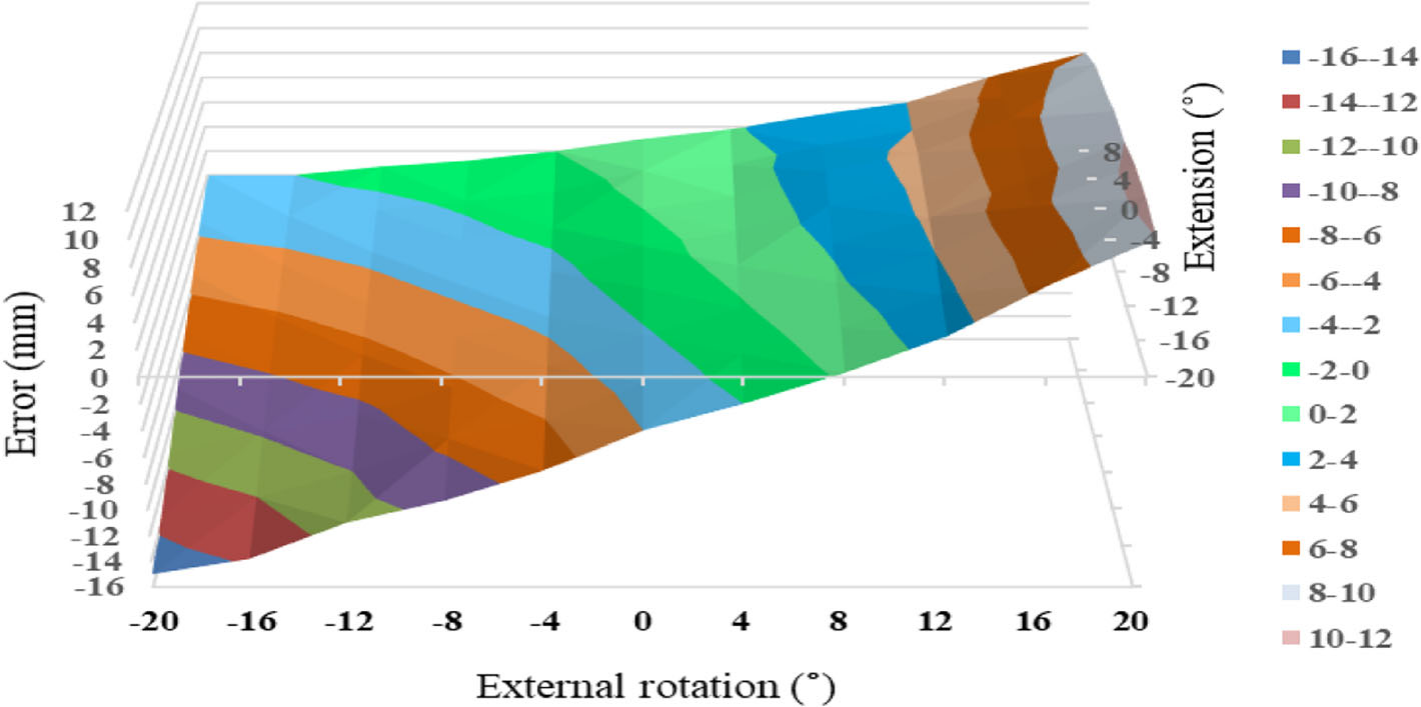
Leg length measurement error in relation to combined limb position for P1 at 0°of adduction. The limb is moved from 20° flexion to 8° extension combined with 20° of internal and external rotation. The permissible range (leg length measurement error within ±2 mm) is painted green

**Fig. 6:**
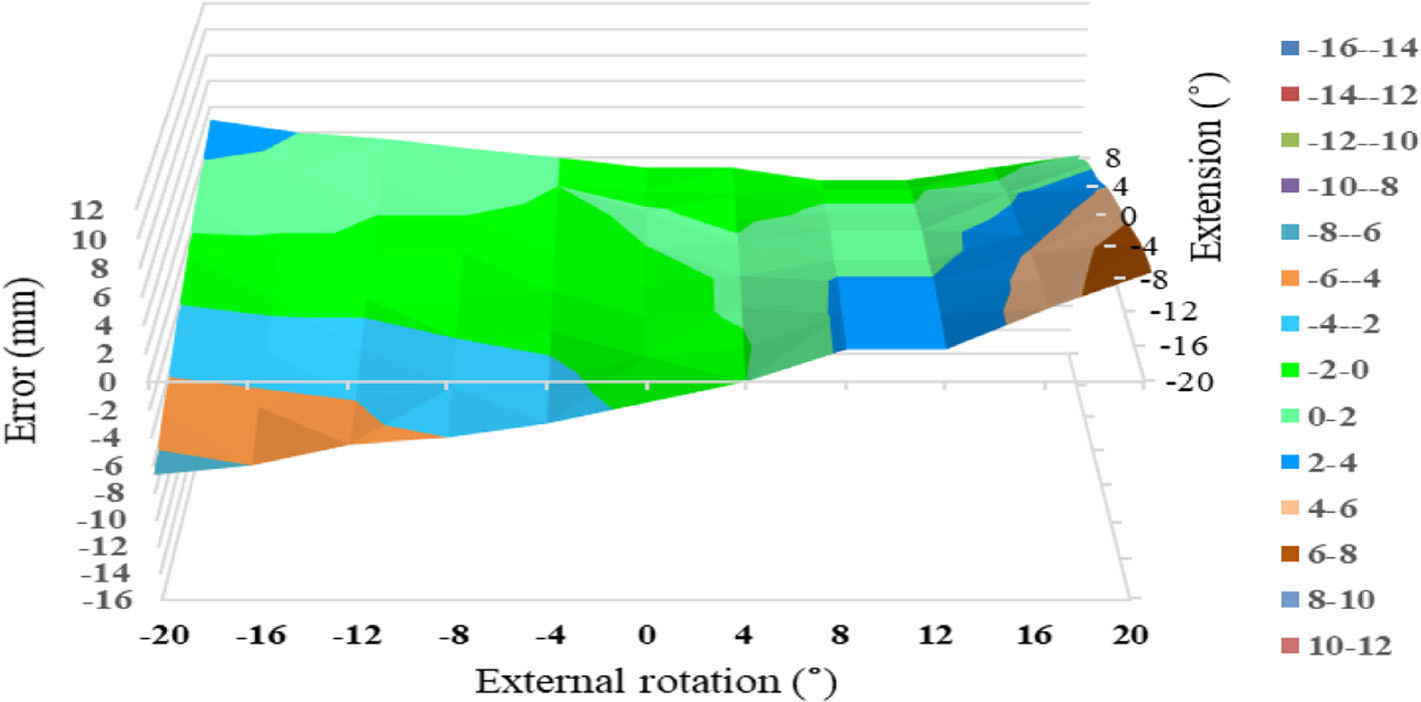
Leg length measurement error in relation to combined limb position for P2 at 0°of adduction. The limb is moved from 20° flexion to 8° extension combined with 20° of internal and external rotation. The permissible range (leg length measurement error within ±2 mm) is painted green

At 0° of ADD, the permissible range of combined limb position for LL was much wider for P2 than for P1 (Figs. 5 and 6). The permissible range of LL for P2 ranged from 16° of IR to 12° of ER combined with 8° of FX and EX. The permissible range of LL for P1 was narrow; for example, only from 4° of IR to 4° of ER was permissible in 0° of FX/EX. In P2, LL error within ±2 mm was seen in 57 of 88 measurement points. In contrast, LL error within ±2 mm was seen in 24 points in P1. LL error within ±2 mm was more frequently observed in P2 than in P1 (chi-square test, *P* < 0.01). At 4° of ADD, the permissible range of LL was much reduced and shifted to FX and IR: LL error within ±2 mm was seen in 17 and 6 points, respectively, for P1 and P2.

**Fig. 7:**
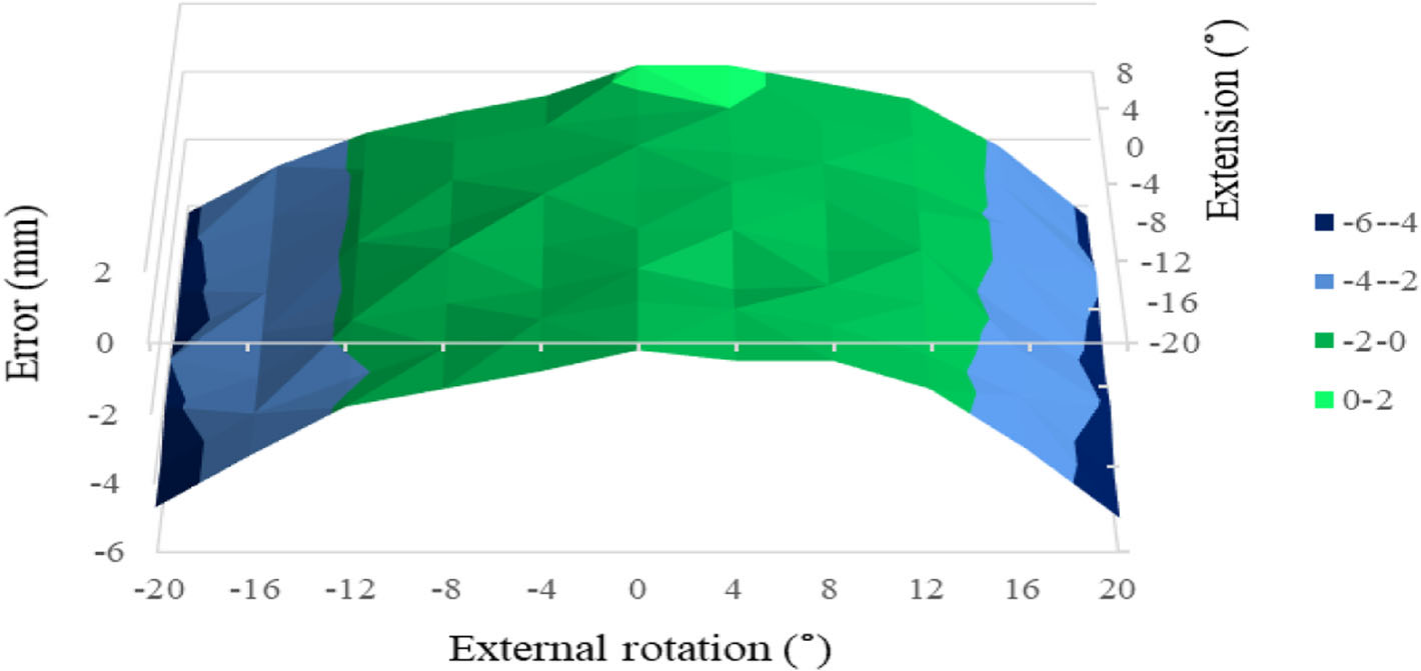
Offset measurement error in relation to combined limb position for P1 at 0° of adduction. The limb is moved from 20° flexion to 8° extension combined with 20° of internal and external rotation. The permissible range (offset measurement error within ±2 mm) is painted green

**Fig. 8:**
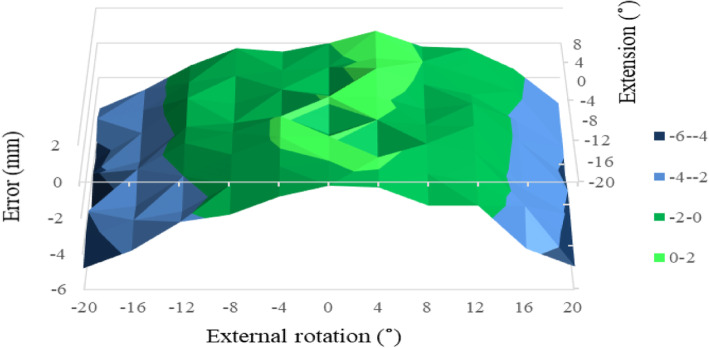
Offset measurement error in relation to combined limb position for P2 at 0° of adduction. The limb is moved from 20° flexion to 8° extension combined with 20° of internal and external rotation. The permissible range (offset measurement error within ±2 mm) is painted green

As for OS, the permissible range of combined limb position at 0° of ADD was wide for both P1 and P2 (Figs. 7 and 8). The permissible range of OS spread from 12° of ER to 12° of IR combined with 16° of FX to 8° of EX. OS error within ±2 mm was seen in 55 and 56 points, respectively, in P1 and P2 (chi-square test, *P* = 0.98). However, the permissible range of combined limb position in OS disappeared totally at 4° of ADD for both P1 and P2.

## Discussion

In the past, several authors described intra-operative LL measurement using callipers, although few authors mentioned intra-operative OS measurement during THA [6, 11, 12]. It is known that precise lower limb repositioning, especially ABD/ADD, is essential to minimize LL and OS measurement errors [13]. To date, no study has investigated the effects of FX/EX and IR/ER on LL and OS measurements. Furthermore, no study has investigated the permissible range of combined limb position for accurate LL and OS measurement.

The purpose of this study was to (1) clarify the effects of lower limb position (FX/EX, ABD/ADD, and IR/ER) on LL and OS errors and (2) determine the permissible range of combined limb position in relation to iliac pin position. In the present study, two iliac pin positions were tested. Although P1 is used in actual THA to prevent pin loosening during surgery, P1 is not the ideal pin position from the perspective of leg length measurement accuracy, because P1 is situated 22° anterior in relation to the centre of rotation of the hip [8, 10, 12]. In contrast, P2 has no anterior or posterior bias in relation to the centre of rotation of the hip, and it would be difficult to achieve secure pin fixation because of the thin and weak nature of living bone near the top of the iliac crest. It was found that changes in limb ABD/ADD had the greatest effect, irrespective of whether the pelvic reference pin was placed in the iliac tubercle (P1) or in the iliac crest in line with the long axis of the femur (P2). ABD or ADD of just 4° resulted in an error of approximately 5–7 mm for LL and 2–4 mm for OS. Sarin et al., using a different technique, obtained results consistent with the present results. They found that 10° of hip ABD or ADD resulted in a 14–17-mm error for LL and 9.5–11.1-mm error for OS [13]. Although the experimental conditions were not identical, both studies showed that limb position in ABD/ADD should be strictly maintained during LL and OS measurement in THA.

In the present study, LL measurement error in association with FX/EX and IR/ER limb position change was smaller for pelvic-pin placement in P2 than for pin placement in P1. Shiramizu et al. developed an L-shaped calliper that enables LL measurement parallel to the limb lengthening axis [12]. They concluded that the error between the intra-operative measurement and the radiographic measurement was reduced significantly in absolute value using the device. Their limb lengthening axis was equivalent to the present line with the longitudinal axis of the femur. Therefore, the present findings support their results. P2 should be chosen as the reference pin position in the ilium to reduce LL measurement error.

Unlike LL, no effect of iliac pin position on OS error was found for FX/EX positional change. Theoretically, OS does not change in FX/EX because the movement of FX/EX takes place in the sagittal plane. The result of the present experiment, that the error of OS measurement in FX/EX was within 1.5 mm for both P1 and P2, was consistent with theory. Although the effect of IR/ER on OS error was 10 mm in total and should not be ignored, the OS error was no more than 2 mm from 12° of IR to 12° of ER.

In actual THA, lower limb reposition could change in combination with ABD/ADD, FX/EX, and IR/ER. The present study showed the permissible range of combined limb position that enables LL and OS errors within ±2 mm for different pelvic pin positions. Without regard to combined FX/EX and IR/ER limb positions, as little as 4° of ADD reduced the permissible range of combined limb position for LL and eliminated that for OS. If ABD/ADD was fixed to neutral (i.e. 0° of ABD/ADD), the permissible range of combined FX/EX and IR/ER limb position in LL for P2 was wide. A serious disadvantage for pelvic reference position P1 is the narrow permissible range near the neutral position. On the other hand, a big advantage for pelvic reference position P2 is the wide permissible range around the neutral position. Because LL and OS measurement during actual THA is done with the lower limb position as neutral as possible [12], pelvic reference position P2 should be chosen to reduce intraoperative LL measurement error. As for OS, the permissible range of combined FX/EX and IR/ER limb position was wide as long as ABD/ADD was fixed to neutral, irrespective of pelvic pin position. Therefore, FX/EX and IR/ER limb positions would not be so critical for OS measurement during actual THA.

In actual THA surgery in the lateral decubitus position, proper attention must be given to maintaining ipsilateral lower limb positioning with regard to ADD/ABD, FX/EX, and IR/ER whenever LL and OS measurements are done. To achieve this condition, we put the ipsilateral lower limb on a sterile Mayo stand with the knee flexed to maintain the neutral ABD/ADD and IR/ER position. Thus, the thigh and the lower leg are held parallel to the floor. Furthermore, we draw the position of the ipsilateral lower limb on the surgical drape overlying the Mayo stand at the beginning of the operation and fit the lower limb within this drawing in order to maintain the FX/EX position.

There are limitations to this study. In this study, an experimental THA simulator in the lateral decubitus position was used. Therefore, all of these results are only applicable to lateral decubitus approaches. In an actual THA operation, not only lower limb but also pelvic position might change during surgery [6, 14]. It is difficult to assess subtle changes in pelvic tilt during THA. However, pelvic tilt might be able to be identified by the change in inclination of the fixed pin on the pelvis from the initial position. Furthermore, the soft tissue factor was not considered. Therefore, all of the results might not be applicable to actual THA. Finally, the LOMI should be improved to measure LL from the pelvic pin position in the iliac crest in line with the longitudinal axis of the femur. A clinical study is necessary to verify the accuracy of LL measurement using the improved device.

Recently, computer navigation systems have been developed for more precise THA surgery [15–17]. Computer navigation provides real-time information about the positional changes of the pelvis and femur. Therefore, computer navigation systems enable accurate LL and OS measurements if relative positional change between the pelvis and the femur is present. However, even in computer-navigated THA, repositioning of the femur is an issue; Renkawitz et al. recommended keeping FX/EX, ABD/ADD, and IR/ER within ±5° compared with the preoperative neutral position [18]. Several studies have shown that more accurate LL and OS reconstruction can be achieved in THA by using the computer navigation system rather than the conventional, non-navigated surgery [19–23]. However, Ogawa et al. showed good equalization of the leg lengths using both computed tomography-based navigation and the simple manual measurement calliper [24]. Many studies pointed out the disadvantages of computer navigation surgery, including the high costs of acquisition and maintenance of the necessary devices, the longer operation time, and the complications related to the pins. Moreover, the accuracy of LL and OS measurements using computer navigation systems is variable [19, 21, 25–28]. In most studies, the accuracy of LL and OS measurements was reported based on the difference between the normal side and the THA side using a postoperative radiograph. The difference is usually set to 4–6 mm, although whether the 4–6 mm difference is sufficient is controversial. In some studies, cases of DDH with preoperative severe subluxation were excluded [23–27]. Accurate intraoperative LL and OS measurements having marked leg length discrepancy might be still challenging even when using a computer navigation system. Therefore, direct LL and OS measurement with LOMI would help surgeons to choose optimal neck options of the femoral stem and the modular femoral heads having different neck length during THA.

## Conclusions

The effects of lower limb position (FX/EX, ABD/ADD, and IR/ER) on LL and OS errors in relation to iliac pin position and the permissible range of combined limb position to achieve measurement errors within ±2 mm of LL and OS were clarified. Because changes in limb ABD/ADD position had the greatest effect on LL and OS measurements irrespective of pelvic pin position, ABD/ADD position should be strictly maintained whenever LL and OS are measured. The pelvic pin position in the iliac crest in line with the longitudinal axis of the femur is preferred, because LL measurement error is reduced, and the permissible range of combined limb position is wide. All this basic information is useful to improve LL and OS measurement accuracy using manual callipers in THA.

## Data Availability

The datasets used and/or analysed during the current study are available from the corresponding author upon reasonable request.

## Abbreviations

THA: Total hip arthroplasty
LL: Leg length
OS: Offset
FX: Flexion
EX: Extension
ABD: Abduction
ADD: Adduction
ER: External rotation
IR: Internal rotation
P1: Pelvic pin position in the iliac tubercle
P2: Pelvic pin position in the iliac crest in line with the longitudinal axis of the femur
DDH: Developmental dysplasia of the hip

## References

[1] Li J, McWilliams AB, Jin Z (2015). Unilateral total hip replacement patients with symptomatic leg length inequality have abnormal hip biomechanics during walking. Clin Biomech.

[2] Sakalkale DP, Sharkey PF, Eng K (2001). Effect of femoral component offset on polyethylene wear in total hip arthroplasty. Clin Orthop Relat Res.

[3] Upadhyay A, York S, Macaulay W (2007). Medical malpractice in hip and knee arthroplasty. J Arthroplasty.

[4] McWilliams AB, Douglas SL, Redmond AC (2013). Litigation after hip and knee replacement in the National Health Service. Bone Joint J.

[5] Röder C, Vogel R, Burri L (2012). Total hip arthroplasty: leg length inequality impairs functional outcomes and patient satisfaction. BMC Musculoskelet Disord.

[6] Ranawat CS, Rao RR, Rodriguez JA (2001). Correction of limb-length inequality during total hip arthroplasty. J Arthroplasty.

[7] Renkawitz T, Weber T, Dullien S (2016). Leg length and offset differences above 5 mm after total hip arthroplasty are associated with altered gait kinematics. Gait Posture.

[8] Dolhain P, Tsigaras H, Bourne RB (2002). The effectiveness of dual offset stems in restoring offset during total hip replacement. Acta Orthop Belg.

[9] Little NJ, Busch CA, Gallagher JA (2009). Acetabular polyethylene wear and acetabular inclination and femoral offset. Clin Orthop Relat Res.

[10] Bourne RB, Rorabeck CH (2002). Soft tissue balancing: The hip. J Arthroplasty.

[11] Barbier O, Ollat D, Versier G (2012). Interest of an intraoperative limb-length and offset measurement device in total hip arthroplasty. Orthop Traumatol.

[12] Shiramizu K, Naito M, Shitama T (2004). L-shaped caliper for limb length measurement during total hip arthroplasty. J Bone Joint Surg [Br].

[13] Sarin VK, Pratt WR, Bradley GW (2005). Accurate femur repositioning is critical during intraoperative total hip arthroplasty length and offset assessment. J Arthroplasty.

[14] Jinjun Z, Zhinian W, Dorr LD (2010). Quantification of pelvic tilt in total hip arthroplasty. Clin Orthop Relat Res.

[15] Renkawitz TR, Sendtner E, Schuster T (2014). Femoral pinless length and offset measurements during computer-assisted, minimally invasive total hip arthroplasty. J Arthroplasty.

[16] Dorr LD, Malik A, Wan Z (2007). Precision and bias of imageless computer navigation and surgeon estimates for acetabular component position. Clin Orthop Relat Res.

[17] Parratte S, Argenson JN (2007). Validation and usefulness of a computer-assisted cup positioning system in total hip arthroplasty. A prospective, randomized, controlled study. J Bone Joint Surg Am.

[18] Renkawitz T, Schuster T, Grifka J (2010). Leg length and offset measures with a pinless femoral reference array during THA. Clin Orthop Relat Res.

[19] Ellapparadja P, Mahajan V, Atiya S (2016). Leg length discrepancy in computer navigated total hip arthroplasty - how accurate are we?. Hip Int.

[20] Licini DJ, Burnikel DJ, Meneghini RM (2013). Comparison of limb-length discrepancy after THA: with and without computer navigation. Orthopedics.

[21] Paprosky WG, Muir JM (2016). Intellijoint HIP®: a 3D mini-optical navigation tool for improving intraoperative accuracy during total hip arthroplasty. Med Devices.

[22] Manzotti A, Cerveri P, De Momi E (2011). Does computer-assisted surgery benefit leg length restoration in total hip replacement? Navigation versus conventional freehand. Int Orthop.

[23] Grosso P, Snider M, Muir JM (2016). A smart tool for intraoperative leg length targeting in total hip arthroplasty: a retrospective cohort study. Open Orthopaed J.

[24] Ogawa K, Kabata T, Maeda T (2014). Accurate leg length measurement in total hip arthroplasty: a comparison of computer navigation and a simple manual measurement device. Clin Orthop Surg.

[25] Ellapparadja P, Mahajan V, Deakin AH (2015). Reproduction of hip offset and leg length in navigated total hip arthroplasty: how accurate are we?. J Arthroplasty.

[26] Renner L, Janz V, Perka C (2016). What do we get from navigation in primary THA?. EFORT Open Rev.

[27] Weber M, Woerner M, Springorum R (2014). Fluoroscopy and imageless navigation enable an equivalent reconstruction of leg length and global and femoral offset in THA. Clin Orthop Relat Res.

[28] Dastane M, Dorr LD, Tarwala R (2011). Hip offset in total hip arthroplasty. Quantitative measurement with navigation. Clin Orthop Relat Res.

